# Dens evagination and complete invagination in the same tooth with extraoral fistula: A rare case report

**DOI:** 10.1002/ccr3.9247

**Published:** 2024-08-07

**Authors:** Behnam Bolhari, Sholeh Ghabraei, Faranak Noori, Nasim Hashemi

**Affiliations:** ^1^ Department of Endodontics, School of Dentistry Tehran University of Medical Sciences Tehran Iran

**Keywords:** cortical bone perforation, cutaneous fistula, dens envaginatus, dens in dent, dens invaginatus, periapical lesion

## Abstract

**Key Clinical Message:**

Successful management of a rare case involving both dens evaginatus and dens invaginatus in the same tooth, monitored over a 24‐month follow‐up.

**Abstract:**

Dens invaginatus (DI) is a congenital dental anomaly characterized by the presence of a tooth that resembles a “tooth within a tooth.” Conversely, dens evaginatus (DE) is a developmental anomaly distinguished by an additional tubercle or cusp on the tooth's crown. Both DI and DE can result in pulpal and periapical diseases in the affected tooth. This article presents a case of a healthy 14‐year‐old male with recurrent swelling under the chin and a wound with pus drainage on the right side of the submental area, associated with his left mandibular lateral incisor affected by both DI and DE. Clinical and radiographic examinations revealed that the tooth was necrotic and had a chronic apical abscess. Cone beam computed tomography (CBCT) confirmed Oehlers' type III DI and a talon cusp on the lingual surface of the same tooth. The patient underwent orthograde endodontic treatment. Passive ultrasonic activation of a 5.25% NaOCl solution and a mixture of Ca(OH)_2_ and a 2% chlorhexidine solution were utilized to effectively clean and eliminate the persistent pus discharge. After the resolution of the patient's symptoms, the apical third of the root canal and the invaginated space were filled with a plug of mineral trioxide aggregate (MTA), while the remaining root canal was filled using a sealer‐based obturation technique. A 24‐month follow‐up visit revealed complete bone regeneration in the previously affected periradicular tissues.

## Graphical abstract

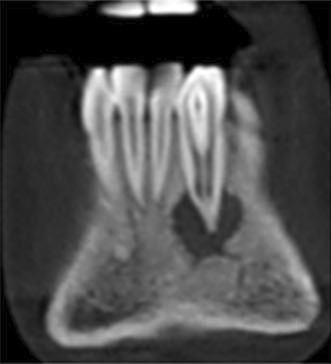

## 1 INTRODUCTION

Dens invaginatus (DI) is a congenital abnormality characterized by the inward folding of the enamel organ into the dental papilla during soft tissue growth. As a result, a miniature tooth‐like structure is formed within the pulp space upon the mineralization of dental tissue.^1^ Oehlers proposed a widely recognized classification for this malformation, which is primarily based on the depth of the defect in relation to the cemento‐enamel junction (CEJ) and its communication with the periodontal ligament (PDL). Type I refers to cases where the defect is limited to the crown of the tooth; Type II involves a defect that extends beyond the CEJ; Type III‐A is characterized by the presence of a pseudo‐canal that connects to the periodontium; and lastly, Type III‐B describes an invagination that penetrates through the root and connects with the periodontal ligament at the apical foramen.^2^ Dental X‐rays can effectively detect different types of DI.

In intraoral radiography, Type I manifests as a deep fissure that extends from the crown toward the pulp; Type II presents as a drop‐shaped radiolucency within the tooth, surrounded by a thin radiopaque line that resembles enamel opacity. Type III, however, mimics an invagination that resembles a canal running parallel to the main root canal, ultimately terminating in the periodontium. Nevertheless, cone beam computed tomography (CBCT) is highly recommended for its precise determination of the specific type of malformation and its ability to assess the communication between the invagination and the root canal system, as well as the periodontium.^3^, ^4^

This anomaly could potentially subject the affected tooth to clinical complications, as the invaginated structure might provide a pathway for the entry of microorganisms. This defect is usually protected by a thin layer of enamel, and it has been observed that the dentin beneath may have small canals. These canals enable communication between the invagination area and the dental pulp, thereby increasing the vulnerability of these teeth to pulpal diseases during the initial stages of tooth eruption.^5^, ^6^

There are two primary categories for treating teeth with dens invagination (DI): those without any associated pathosis, and those with accompanying pathosis. In cases where there is no pathosis, it is advisable to perform prophylactic sealing, provide oral hygiene instructions, and regularly monitor the condition. However, for cases where irreversible pulpitis or pulpal necrosis is present, treatment of the root canal system becomes crucial. Ensuring complete cleaning, disinfection, and obturation of the canal poses a significant challenge in such situations.^7^, ^8^

Dens evaginatus (DE) is a congenital abnormality characterized by the protrusion of the enamel organ, resulting in the formation of an extra cusp. This additional cusp is typically found in the central groove or ridge of posterior teeth and in the cingulum area of anterior teeth.^9^ It is worth noting that DE and talon cusp are terms that can be used interchangeably to describe the same dental anomaly.

Talon cusp, a specific type of DE, commonly manifests on the lingual surface of anterior teeth. Hattab et al. have further classified talon cusp into three distinct types: Type 1, known as Talon, refers to a cusp that extends at least halfway from the CEJ to the incisal edge; Type 2, referred to as Semi‐talon, is characterized by an additional cusp of at least 1 mm in length, which extends less than the distance from the CEJ to the incisal edge; Type 3, known as Trace talon, is distinguished by an enlarged or prominent cingulum, along with various manifestations such as conical, bifid, or tubercle‐like structures.^10^

Clinical complications associated with DE include the possibility of tubercle fracture or wear, occlusal interference, attrition, tongue irritation, plaque accumulation, and dental caries.^11^ To prevent the emergence of symptoms, different prophylactic treatments are recommended. When managing talon cusp, careful clinical decision‐making is crucial.

Treatment options for this condition involve the application of resin to strengthen the tubercles, the placement of prophylactic restorations, selective grinding of the tubercles, extraction, and partial pulpotomy.^12^

Caries, dental anomalies, trauma, and subsequent pulpal necrosis are the main factors contributing to the development of an extraoral sinus tract of odontogenic origin in the face and neck region. The odontogenic infection has the potential to spread widely through the surrounding tissues along fascial planes.^13^ Dental examinations should be considered a crucial method for accurately diagnosing the underlying cause, especially when there is no swelling or pain in the mouth, which could lead to the misdiagnosis and improper treatment of chronically draining cutaneous sinus tracts. This is particularly relevant when the teeth are intact.^14^

This article presents the successful management of a rare case involving a cutaneous fistula on the right side of the submental area and a large periapical lesion resulting in a perforation of the buccal bone cortex, related to the left mandibular lateral incisor, which was affected by both DI and DE.

## 2 CASE HISTORY

A healthy 14‐year‐old male with a noncontributory medical history was referred to the Department of Endodontics at the School of Dentistry, Tehran University of Medical Sciences. He presented with a chief complaint of recurrent submental swelling and a wound with purulent drainage on the right side of the chin.

## 3 DIAGNOSIS

**FIGURE 1:**
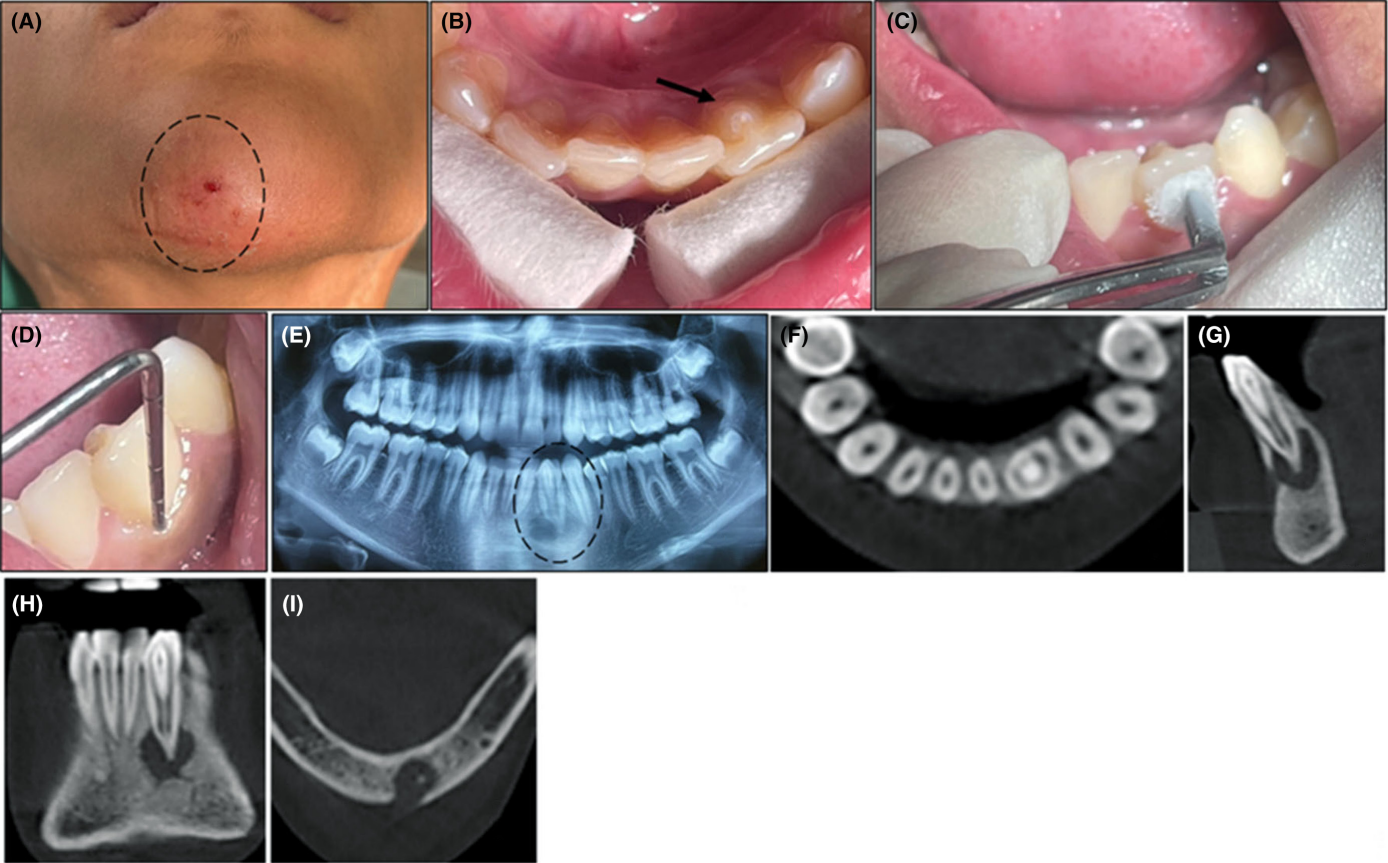
(A) An extra oral fistula in right side of the chin. (B) Talon cusp. (C) Cold sensibility test revealed tooth necrosis. (D) Probing depth was in normal limited. (E) Panoramic radiograph shown a large periapical lesion in left mandibular lateral incisor affected by dens invagination anomaly. (F) CBCT, axial view. (G) CBCT, coronal view. (H) CBCT, sagittal view. (I) CBCT, axial view demonstrated cortical bone perforation.

During the initial extraoral examination, swelling and a bleeding fistula were noted (Figure 1A). The intraoral examination revealed good oral hygiene and normal occlusion with no missing teeth or caries. The palatal surface of the mandibular left lateral incisor exhibited a talon cusp (Figure 1B). This tooth did not respond to a sensibility test and was not tender to percussion and palpation (Figure 1C). Probing depths of all anterior teeth were within the normal range (Figure 1D). The diagnostic periapical radiograph revealed (Figure 1E). The root canal configuration of this tooth corresponded with Oehlers' type III‐B DI. A CBCT scan was recommended for the mandibular left lateral incisor to better define and visualize the exact extent of the periapical lesion and the morphology of the root canal system. The CBCT evaluation identified the extent of the invagination, demonstrating its connection with the periodontium at the apical foramen. Two separate canals with distinct apical foramina were observed: one inside the DI and a main canal around the inner tooth. The associated periapical lesion had perforated the buccal cortical bone (Figure 1F–I). Based on clinical and radiographic examinations, the diagnosis for the mandibular left lateral incisor was pulpal necrosis and a chronic apical abscess.

## 4 TREATMENT

**FIGURE 2:**
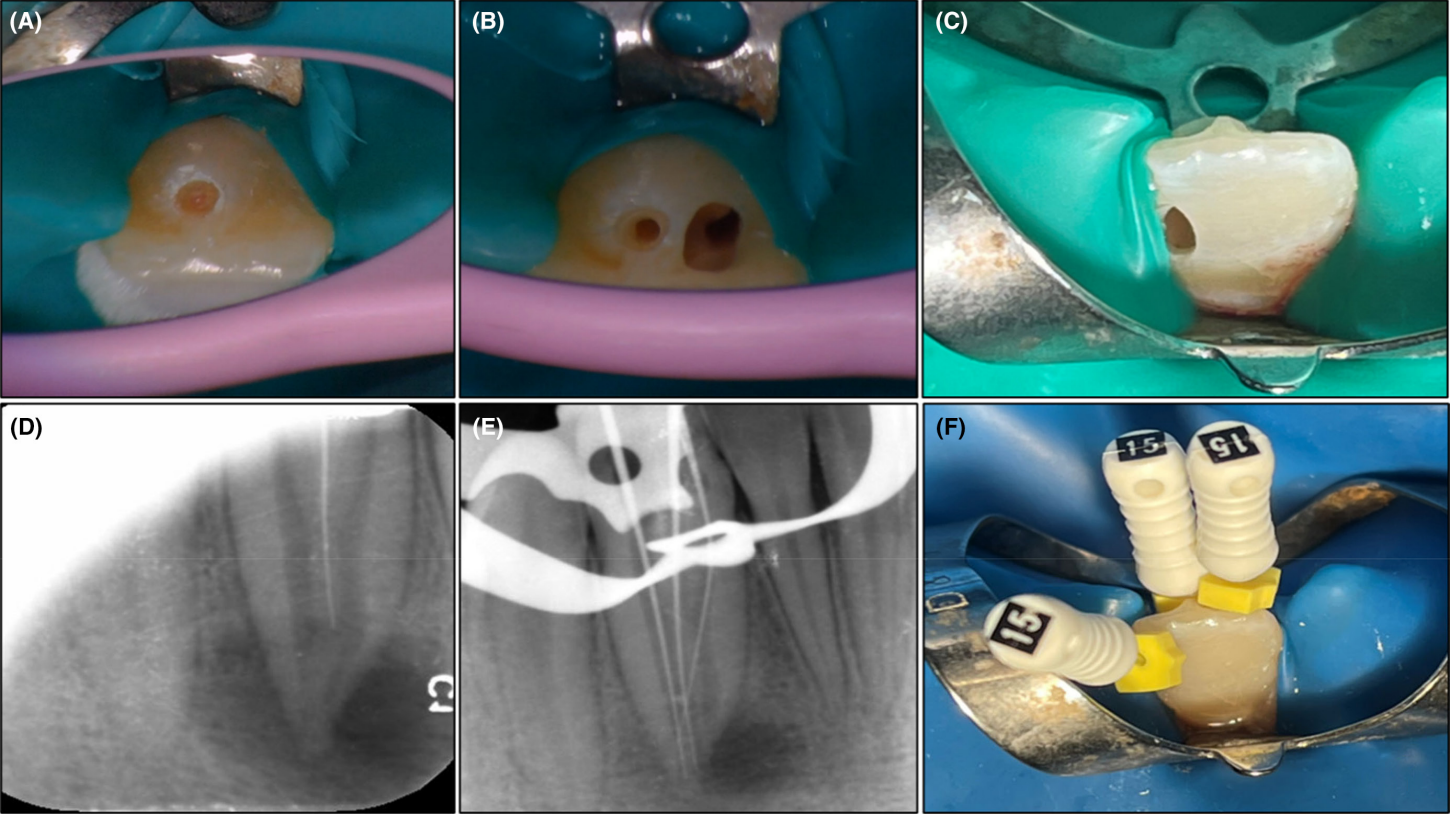
(A) First access cavity in the tip of talon cusp. (B) Second access cavity in distolingual surface of the tooth crown. (C) Third access cavity in mesiobuccal surface of the tooth crown. (D) Exploration of the canals with a #10 C‐pilot file. (E, F) Confirming working length by radiography.

After obtaining informed consent, root canal treatment was initiated. At the first treatment visit, local anesthesia (Xylopen 2%, Exsir, Iran) was administered. A comprehensive review of the CBCT was conducted and the tooth was isolated with a rubber dam. An access cavity was then prepared from the tip of the talon cusp aiming to reach the inner tooth canal space (Figure 2A). To access the main root canal, a second access cavity was prepared on the distolingual surface of the tooth crown (Figure 2B). Additionally, due to teeth crowding and lack of access to the mesiolingual surface of the tooth crown, an access cavity was prepared on the mesiobuccal surface to reach the part of the main canal located in the mesial area of the DI (Figure 2C). These steps were performed under 6× magnification using a dental operating microscope (DOM) (Carl Zeiss, Oberkochen, Germany). During the exploration with a #10 C‐pilot file (VDW, Munich, Germany) in each access cavity, and with assistance from the DOM, it was noted that a pathway extended from the apical area of the inner tooth to the apical foramen. This pathway exhibited calcification and appeared like a calcified canal and prepared like a calcified canal (Figure 2D). Subsequently, three #15 K‐files (VDW, Munich, Germany) were placed in all three pathways, and their positions were confirmed using an electronic apex locator (Root AZ, J. Morita, Japan) and periapical radiographs (Figure 2E,F).The main root canal and the inner tooth canal space were prepared using the crown‐down technique with rotary NiTi instruments (DENCO Super Files III, Longhua, Shenzhen, China). A 5.25% NaOCl solution was used as the root canal irrigant between each instrumentation step, agitated with an ultrasonic device (Varios 970, NSK, Japan). Following this, a calcium hydroxide dressing was placed within the canal, and the access cavities were sealed with temporary restoration material (Morvabon Z.O.E Cement, Iran).

**FIGURE 3:**
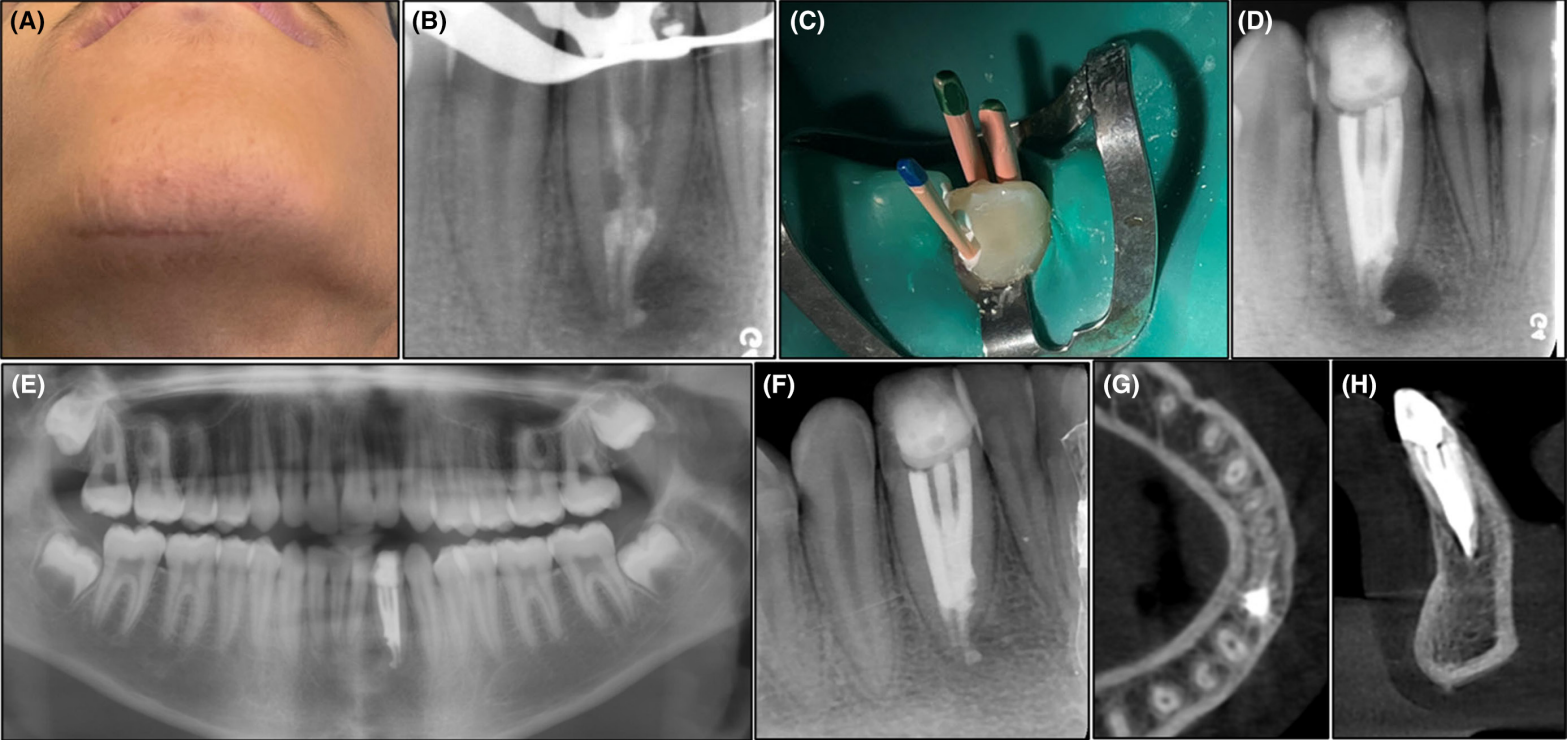
(A) Swelling of the chin and the fistula were resolved. (B) Apical plug with MTA. (C, D) Obturation the canals by EndoSeal MTA with single con technique. (E) 12‐month follow‐up. (F) Panoramic radiograph after 24‐months shown complete bone heling. (G, H) CBCT revealed Complete healing of the periradicular lesion and formation of a bone cortex.

Two weeks later, at the second visit, the swelling on the right side of the chin and the fistula had resolved (Figure 3A). The temporary filling was removed, and the affected tooth was isolated with a rubber dam. Under magnification with a DOM, the calcium hydroxide was carefully removed. The canal was thoroughly disinfected using a 5.25% sodium hypochlorite solution, activated with ultrasonic. Due to external resorption at the apical part of the root, RetroMTA (BioMTA, Daejeon, Seoul, Korea) was placed as an apical plug to provide an adequate seal in the apical portion of the root canal (Figure 3B). A moist paper cone was placed over the MTA to ensure proper setting, followed by a temporary restoration.

During the third visit, 2 days later, the setting of the MTA was confirmed. To achieve 3‐dimensional (3D) obturation of the remaining portion of the canal, a flowable bioceramic sealer (Marruci EndoSeal MTA, USA) was used. The sealer was agitated using an ultrasonic device to ensure proper distribution and adaptation to the dentinal walls. Subsequently, three #0.06 taper gutta‐percha cones were placed in the canals through the three access cavities (Figure 3C,D). The patient was then referred for tooth restoration, and the affected tooth was restored using a composite resin adhesive.

## 5 FOLLOW‐UP

Following a periapical radiographic evaluation after 12 months, complete bone formation was observed in the periradicular region, and the patient's clinical examination indicated the absence of any signs or symptoms (Figure 3E). After a duration of 24 months, the radiographic examination revealed complete healing of the teeth and surrounding bone, along with the formation of a bone cortex (Figure 3F–H).

## 6 DISCUSSION

Teeth exhibiting DE and DI anomalies are more susceptible to early pulpal pathosis.^15^, ^16^ Therefore, early diagnosis and subsequent preventive treatment are recommended. Due to the absence of caries‐related pulp pathosis in these teeth, patients often remain unaware of any dental issues. Consequently, early diagnosis of this condition is frequently overlooked, leading patients to seek treatment only when pulp and periapical disease have progressed to advanced stages.^17^, ^18^ In the present case report, the patient had no decay in the affected teeth, and no other anomalies were observed in the remaining teeth. Additionally, there was recurrent submental swelling and a wound with purulent drainage on the opposite side of the affected teeth. In children and young adolescents, the alveolar process is not fully developed, increasing the risk of odontogenic infections spreading beyond muscle attachments and causing a cutaneous sinus tract. These infections can also occur in areas distant from the affected tooth.^19^

The joint position statement from the American Association of Endodontists (AAE) and the American Academy of Oral and Maxillofacial Radiology (AAOMR) recommends the use of Cone Beam Computed Tomography (CBCT) in cases with complex root canal anatomy, such as DI.^20^ The information obtained through CBCT can greatly enhance the clinician's ability to manage such cases.^21^ Treatment decisions for teeth with DI should be based on the DI classification and the pulpal and periapical condition. If the tooth becomes necrotic, the invagination and the root canal system can be treated and obturated separately.^18^

In the current case, given the extent of the invagination, the final diagnosis was established as Oehlers' Class III DI, accompanied by pulpal necrosis and a large periapical lesion. The periapical diagnosis was a chronic apical abscess. The treatment plan consisted of cleaning the invaginated space and performing root canal treatment on the main tooth. Since the CBCT evaluation revealed that the invaginated defect resembled a complete tooth within the main root canal, it was decided to treat the inner tooth canal space and the main root canal separately. Ultrasonic activation was used to remove all necrotic tissue and bacterial biofilm, ensuring adequate debridement of the irregularities in the invagination and root canal system.^22^ Use of intracanal medicaments between treatment sessions have been advocated by some investigators especially when there is active discharge within the root canal.^23^ Calcium hydroxide is the commonly used medicament that is proven to have antibacterial activity against common endodontic pathogens. This medicament affects bacteria through damage to the bacterial cytoplasmic membrane, protein denaturation and damage to the DNA.^24^, ^25^, ^26^, ^27^ When pulp necrosis occurs, it triggers an inflammatory reaction in the tissues surrounding the root. This inflammation leads to the resorption of both the apical portion of the root and the adjacent bone. Without treatment, this resorption progresses, damaging the apical foramen and apical constriction.^28^

In situations where the apex is open, either because the tooth is not fully mature or due to root resorption, employing calcium silicate cements as an apical plug is a treatment option to provide an adequate seal in the apical portion of the root canal.^29^ In this case, due to apical destruction caused by inflammatory root resorption, it was decided to use an MTA apical plug to create a barrier at the apex of the root. Obturation of the root canal in teeth with DI is very challenging. Due to the complexity and irregularity of the root canal in teeth with DI, some studies recommend using thermoplasticized gutta‐percha to ensure complete filling of the root canal space. Additionally, other studies suggest filling the entire canal space with Mineral Trioxide Aggregate (MTA) cement.^30^, ^31^ In the present case, a 4 mm MTA plug was placed in the apical area, and the remaining portion of the canals was filled using a sealer‐based obturation technique with Endoseal MTA and a single‐cone gutta‐percha. In cases with DI, due to the irregularity and multiple paths inside the root canal, it is important to use a filling material with high flowability to penetrate the irregularities. According to the findings of previous investigations, Endoseal MTA exhibited significantly greater flow values compared to other sealers. Flow is crucial as it enables a sealer to effectively permeate the irregularities and accessory canals within the root canal system. Additionally, a recent study further corroborated the favorable attributes of this particular MTA sealer.^32^, ^33^, ^34^

In our case, after the treatment, the patient was asymptomatic and the radiographic examinations showed complete bone formation and no signs of periradicular pathology during the follow‐up period.

Using CBCT for the initial evaluation and diagnosis of resorption, in addition to two‐dimensional radiography, is essential. However, intraoral periapical radiographs are suitable for continuous follow‐up in accordance with ALARA principles, and usually there is no need to prescribe CBCT,^35^ but in this case, due to the significant perforation of the buccal cortical plate, CBCT were prescribed to verify the full bone formation in the affected area, thus eliminating the necessity for surgical intervention.^36^ Based on the Patel et al.^37^ study, using CBCT is a valuable tool for determining the radiological outcome 1 year after root canal treatment.

Diagnosis and treatment of DI requires a combination of several processes in the diagnostic, preparing the access cavity, disinfection of the root canal system and invagination defect and filling them.

If you can thoroughly clean the root canal system and the invagination, and ensure adequate apical and coronal sealing, there is a possibility of complete healing of the periapical lesion with orthograde nonsurgical treatment, in young patients, even in the presence of cortical bone perforation.

## AUTHOR CONTRIBUTIONS

**Behnam Bolhari:** Conceptualization; data curation; methodology; supervision; visualization. **Sholeh Ghabraei:** Conceptualization; supervision; validation; visualization. **Faranak Noori:** Conceptualization; data curation; methodology; writing – original draft; writing – review and editing. **Nasim Hashemi:** Data curation; methodology; project administration; writing – original draft; writing – review and editing.

## FUNDING INFORMATION

The study had no financial support.

## CONFLICT OF INTEREST STATEMENT

None.

## ETHICS STATEMENT

For clinical cases, the local ethics committee considers that the patient's consent is sufficient.

## CONSENT

Written informed consent was obtained from the patient to publish this report in accordance with the journal's patient consent policy.

## Data Availability

The data supporting the findings of the present study are available from the corresponding author upon request.
